# Cocreative Development of the QoL-ME: A Visual and Personalized Quality of Life Assessment App for People With Severe Mental Health Problems

**DOI:** 10.2196/12378

**Published:** 2019-03-28

**Authors:** David C Buitenweg, Ilja L Bongers, Dike van de Mheen, Hans AM van Oers, Chijs van Nieuwenhuizen

**Affiliations:** 1 Scientific Center for Care and Wellbeing (Tranzo) Tilburg School of Social and Behavioral Sciences Tilburg University Tilburg Netherlands; 2 GGzE Institute for Mental Health Care Eindhoven Netherlands; 3 IVO Addiction Research Institute Erasmus Medical Centre Rotterdam Netherlands; 4 National Institute for Public Health and the Environment Bilthoven Netherlands

**Keywords:** mobile app, quality of life, mental health, homeless persons, medical informatics

## Abstract

**Background:**

Quality of life (QoL) is a prominent outcome measure in mental health. However, conventional methods for QoL assessment rely heavily on language‐based communication and therefore may not be optimal for all individuals with severe mental health problems. In addition, QoL assessment is usually based on a fixed number of life domains. This approach conflicts with the notion that QoL is influenced by individual values and preferences. A digital assessment app facilitates both the accessibility and personalization of QoL assessment and may, therefore, help to further advance QoL assessment among individuals with severe mental health problems.

**Objective:**

This study focused on the development of an innovative, visual, and personalized QoL assessment app for people with severe mental health problems: the QoL-ME.

**Methods:**

This study targeted 3 groups of individuals with severe mental health problems: (1) people with psychiatric problems, (2) people treated in forensic psychiatry, and (3) people who are homeless. A group of 59 participants contributed to the 6 iterations of the cocreative development of the QoL-ME. In the brainstorming stage, consisting of the first iteration, participants’ previous experiences with questionnaires and mobile apps were explored. Participants gave their feedback on initial designs and wireframes in the second to fourth iterations that made up the design stage. In the usability stage that comprised the final 2 iterations, the usability of the QoL-ME was evaluated.

**Results:**

In the brainstorming stage, participants stressed the importance of privacy and data security and of receiving feedback when answering questionnaires. Participants in the design stage indicated a preference for paging over scrolling, linear navigation, a clean and minimalist layout, the use of touchscreen functionality in various modes of interaction, and the use of visual analog scales. The usability evaluation in the usability stage revealed good to excellent usability.

**Conclusions:**

The cocreative development of the QoL-ME resulted in an app that corresponds to the preferences of participants and has strong usability. Further research is needed to evaluate the psychometric quality of the QoL-ME and to investigate its usefulness in practice.

## Introduction

### Background

Quality of life (QoL) has risen to prominence as an outcome in mental health care. Still, many authors agree that there is further room for improvement in the field of QoL assessment, especially regarding the instruments used to assess QoL [1,2]. Several possibilities for advancement have been pointed out in the literature. First, it is important that instruments are frequently updated to maintain their applicability in our fast-paced society. Examples of developments that may influence the meaning of QoL for people with severe mental health problems include an increasing emphasis on empowerment [3-5] and the advancing digitalization of society [6]. Second, research has indicated the need for personalization of QoL instruments, as QoL differs within groups and between individuals [7,8]. This notion calls for a QoL instrument that enables respondents to select and answer questions on domains of QoL that are relevant for them personally. Third, traditional language-based QoL assessment, which relies heavily on people’s verbal and cognitive abilities, might be less appropriate for people with severe mental health problems [9,10]. Visual communication may provide a suitable alternative as it does not require the mastery of a certain language. In addition, visual information may be easier to process by people with severe mental health problems than verbal information [11,12]. Several characteristics of digital technologies make them potentially useful for tackling the aforementioned issues in QoL assessment. A digital instrument has the flexibility to allow for the increased personalization of QoL assessment. In addition, digital technologies facilitate the use of audio and visual multimedia such as images and video, which may improve the accessibility of a digital QoL instrument and help circumvent language-based communication. Furthermore, a digital instrument can easily be updated to incorporate new aspects of QoL that become important as a function of societal changes.

Over the last few years, many digital electronic health (eHealth) technologies for use in mental health care have been developed [13]. People with severe mental health problems use eHealth to obtain information, for Web-based treatment, and as a source of support [13,14]. eHealth for people with severe mental health problems initially focused on the design and development of websites used for treatment, for communication, and to provide information [15-17]. Recently, the rising popularity of mobile devices such as smartphones and tablets has facilitated a shift from websites to mobile health apps for mobile devices such as smartphones and tablets. These mobile health apps have been developed for a variety of psychiatric problems, including anxiety [18], bipolar disorder [19], and schizophrenia [20], and serve a number of purposes, such as treatment, providing information, self-assessment, and self-management [21-25].

Previous studies reveal that websites and apps that are well designed for the general public may not be appropriate for people with severe mental health problems [26-29]. In response to these findings, several authors have reported best practices and guidelines for the design and development of eHealth apps for people with severe mental health problems [21,29-31]. Ben-Zeev et al [30] list a number of specific recommendations for how eHealth apps may best be developed. They stress the importance of working in multidisciplinary teams and involving intended users in the development [30]. Furthermore, Rotondi et al [31] developed the Flat Explicit Design Model (FEDM) to guide the design of eHealth for people with severe mental illness. The model contains 18 variables, grouped into 3 usability dimensions: (1) page complexity, (2) navigational simplicity, and (3) comprehensibility. Examples of variables include minimizing potential distractors, limiting navigational elements, fixing the location of navigational elements, and minimizing page length. Empirical evidence for the usefulness of the FEDM in reducing the cognitive effort for users has been found [31]. These design recommendations are likely to benefit the usability of eHealth technologies for people with severe mental health problems.

### Objectives

This research covers the cocreative development of a QoL assessment app that does not rely solely on language-based communication, facilitates personalization, and is useful for both patients and clinicians: the QoL-ME. The aforementioned design recommendations will be taken into account, but the development of the QoL-ME will primarily be based on the input of end users, which continues to be the standard in design in general [32,33] and in the design for people with severe mental health problems in particular [21,30,34-37]. This study aimed to describe the development of the QoL-ME, with special attention to patients’ design-related preferences.

## Methods

### Participants

This study targeted 3 groups of individuals with severe mental health problems: (1) people with psychiatric problems, (2) people treated in forensic psychiatry, and (3) people who are homeless. Homeless individuals were included in this study because of the high prevalence of severe mental health problems in this group [38-40]. There are several reasons for suspecting that these groups may have difficulty with traditional language-based QoL assessment. First, they experience fewer educational opportunities [38,41,42]. Second, mild intellectual disabilities occur relatively frequently in these groups [38,43,44]. Third, psychopathology itself may compromise individuals’ ability to engage in QoL assessment [9,10].

Participants were recruited with the help of 6 societal institutions that collaborated in a consortium to facilitate this research project, including a mental health institution; a hospital for forensic psychiatry; a multimodal day treatment center for multiproblem young adults; a day center for people who are homeless; and 2 research institutions focusing on lifestyle, homelessness and addiction.

### Development of the QoL-ME

**Figure 1:**
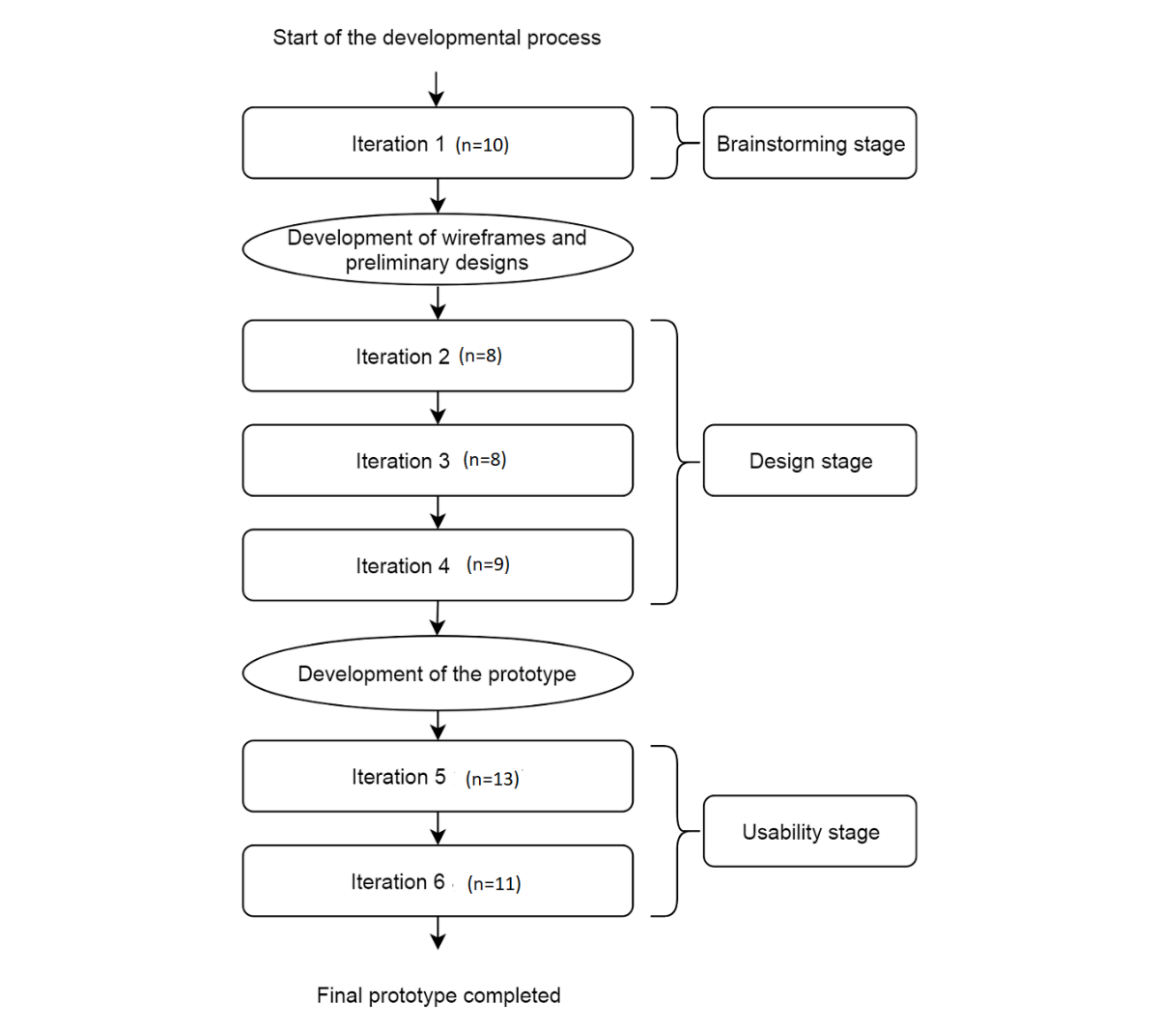
Schematic overview of the development of the QoL-ME, involving 3 stages and 6 iterations.

The QoL-ME was cocreatively developed in an iterative development process in which the 3 aforementioned groups of people with severe mental health problems played an essential and indispensable role. The process consisted of 6 iterations divided over 3 stages: (1) brainstorming stage, (2) design stage, and (3) usability stage. Theoretically, the development process fits in the *explore*, *approximate*, *refine* framework as part of participatory design [45]. A study by Ben-Zeev et al [20] employs a similar approach consisting of 3 steps that correspond to this framework. A schematic overview of the developmental process is provided in Figure 1.

Every iteration involved 3 separate user test sessions, and the total number of test sessions was 18. A new group of participants was recruited in every test session. The 3 target groups were involved in every single iteration. In addition, the age distribution of participants was roughly the same in every iteration. Between 2 and 5 individual participants contributed in every test session. The feedback, tips, and insights of end users gathered during test sessions were of vital importance and were fed back to the professional designers who took care of the technical side of the development. In between iterations, the researchers and professional designers discussed the feedback gathered during the previous iteration. If the end users’ opinions and preferences contradicted each other, an attempt at a synthesis was made during this discussion. If necessary and possible, 2 rivaling preferences were tested in the next iteration. In all stages of the development, the input and opinions of end users were instrumental and were used to expand and refine the initial designs and early versions of the app.

The brainstorming stage involved iteration 1. In this stage, participants were invited to share their past experiences with apps, share ideas regarding the improvement of QoL assessment, and comment on basic initial designs. The topic list that was used during the brainstorming stage is available in Multimedia Appendix 1. On the basis of the ideas that were gathered in this stage, combined with design-related recommendations found in the scientific literature [21,31,35], a number of designs and interaction mechanisms were developed for testing.

The design stage covered iterations 2, 3, and 4. Initially, paper sketches (wireframes) were used to test alternative navigational structures, various possible page-layouts, and possible forms of interaction for the app. In the remainder of the design stage, digitalized versions of these wireframes were gradually refined, expanded, and made functional. Finally, the first prototype was developed.

In iterations 5 and 6, which together formed the usability stage, the prototype was subjected to usability testing. Participants were invited to complete a single task: to fill out the QoL-ME. To test if participants were able to use the prototype independently, no explanation regarding the QoL-ME was provided. The usability of the prototype was systematically assessed using a modified Dutch version of the System Usability Scale (SUS) [46-48].

### Measures

In 7 of the 12 test sessions, participants consented to audio recordings. In the other 5 test sessions, the researchers took extensive notes. The researchers made an elaborate summary of every test session of the first 4 iterations, based on either the recordings or the notes. The summaries included all the participants’ insights, ideas, and feedback and were discussed together with the designers. On the basis of these discussions, the designers elaborated, adjusted, and polished the QoL-ME.

The English version of the SUS was developed by Brooke [47] and has since been used frequently to assess the usability of a variety of technologies such as websites, operating systems, and hardware [48]. The SUS contains 10 items, scored on a 5-point Likert scale ranging from *strongly disagree* (1) to *strongly agree* (5). Its psychometric properties have been investigated by Bangor et al [46], who analyzed the SUS data of 2324 participants and found a Cronbach alpha of .911. In addition, the authors report strong face validity, high sensitivity, and good concurrent validity [46]. The SUS has been translated into several languages, including Dutch [48]. To facilitate people with severe mental health problems, all the items of the Dutch SUS were worded positively in this study, as advised by Sauro and Lewis [49]. In addition, 3 items that contained complex terms were modified slightly without altering their content. Total SUS scores range between 0 and 100. On the basis of the analysis of a large amount of SUS data, scores above 73 are considered to indicate *good* usability, whereas scores above 85 are considered *excellent* [50].

### Procedure

At the start of every test session, the researcher explained the goal of the research project and how participants were invited to contribute. Next, participants gave their informed consent and were asked if they consented to the audio recording of the test session. To prevent acquiescence bias, the researcher emphasized that they did not create the designs or prototypes themselves. In addition, the researcher stressed that there were no right or wrong answers but that participants’ opinions, ideas, and insights counted. In the brainstorming stage, participants were asked a number of questions, after which they were invited to comment on a number of basic initial designs. In the design stage, participants were invited to comment on the layout of the wireframes and to test various forms of interaction and navigation. In the usability stage, participants were invited to use the QoL-ME and to fill out the SUS afterward. At the end of a session, participants were asked if they had any additional feedback, tips, or questions. Moreover, the researchers explained that participants’ feedback was used to refine the designs, and participants received a 10 Euro gift voucher.

All designs and prototypes were tested using an Apple iPad Air 2, which had a 9.7-inch touchscreen display. The researcher provided this iPad.

Ethical approval was obtained from the Ethics Committee of the Tilburg School of Behavioural and Social Sciences at Tilburg University (EC-2015.44). Informed consent was obtained from each participant. All procedures performed in this study involving human participants were in accordance with the ethical standards of the institutional and national research committee and with the 1964 Helsinki Declaration and its later amendments or comparable ethical standards.

### Structure and Content of the QoL-ME

The results of the development of the QoL-ME app are difficult to interpret without additional knowledge of the structure and content of the QoL-ME. To enable an adequate understanding of the results of this study, the conceptual framework underlying the QoL-ME is described in this section.

The QoL-ME consists of 2 main components: a core version and additional modules. The core version comprises a fixed set of universal QoL domains, and every respondent is required to answer questions on these domains. Research indicates that having meaning in life is especially important for people who are homeless [51,52]. The QoL-ME, therefore, encompasses 2 separate core versions. The first core version targets people with psychiatric problems and people treated in forensic psychiatry and includes 3 domains of the Lancashire Quality of Life Profile (LQoLP) [53]: *safety*, *living situation*, and *finances*. A recent study indicates that these 3 LQoLP domains are universal for people with psychiatric problems and people treated in forensic psychiatry [54]. The LQoLP uses a 7-point Likert scale, ranging from *cannot be worse* (1) to *cannot be better* (7). The second core version is tailored to people who are homeless and comprises the Dutch version of the Meaning in Life Questionnaire, a 10-item measure that assesses both the presence of meaning in one’s life and the search for meaning in life [55]. The Meaning in Life Questionnaire also uses a 7-point Likert scale, ranging from *completely disagree* (1) to *completely agree* (7).

The additional modules serve to ensure the personalization of the QoL-ME. Every module corresponds with a domain of QoL, and users are free to select any combination of the 8 modules. The following 8 domains of QoL are included: (1) Support and Attention, (2) Social Contacts, (3) Happiness and Love, (4) Relaxation and Harmony, (5) Leisure, (6) Lifestyle, (7) Finances, and (8) Health and Living. These domains were identified in a visual concept mapping study of the QoL of people with severe mental health problems [56]. Domains are assessed using 2 to 4 visual items. Every visual item contains 3 pictures that together denote an aspect of QoL. Users respond to these items using a visual analog scale (VAS) with visual anchors.

This structure, involving both a core version and additional modules, makes for a flexible QoL assessment app. The core version is useful in contexts where group-level data are of interest, such as comparisons of the QoL of different client populations. The additional modules are especially suitable for use in individual care planning.

## Results

### Participants

A total of 59 participants contributed to the development of the QoL-ME. Their mean age was 40.8 years (SD 15), and over 80% were male (see Table 1). The mean age of the 10 participants who engaged in the brainstorming stage was 34.2 years (SD 12.8), 7 of whom were male. In the design stage, a group of 25 people with severe mental health problems participated. Their mean age was 37.7 years (SD 14.3), and 88% were male. In the usability stage, 79% of the participants (19/24) who contributed were male. Their mean age was 46.8 years (SD 14.4). The number of participants who contributed to the development process is displayed in Table 1.

### Development of the QoL-ME

Participants in the brainstorming stage reported using apps primarily for communication and maintaining social relations. In addition, 4 younger participants treated in forensic psychiatry reported using apps for services such as internet banking and Web-based shopping. The single most important factor for why participants used certain apps over others was their confidence in the trustworthiness of the apps. The majority of participants indicated having privacy concerns when using apps, but this did not seem to deter them from using apps frequently.

All participants had prior experience with questionnaires, primarily in the context of professional care or research. Participants reported several annoyances regarding their previous experiences with questionnaires, 2 of which were relevant for the development of the QoL-ME: (1) lack of feedback and (2) lack of transparency regarding data use. These considerations were fed back into the development of the QoL-ME. In practice, a feedback module that provided users with insight into their scores was implemented, and special consideration was given to the issue of data ownership, leading to the decision that users retain the ownership of their data.

The participants in the brainstorming stage had a number of ideas regarding the QoL-ME. Some participants indicated a preference for the personalization of the app’s appearance by selecting a personal background or by changing the colors of the app. In addition, participants pointed out that not every patient has their own device and therefore advocated a multiplatform app. Furthermore, a combination of visual- and language-based communication was proposed, and some participants even indicated a preference for audio. Whenever possible, these ideas were incorporated into the initial designs of the QoL-ME that were tested in the subsequent iterations.

As displayed in Table 2, the feedback received on the designs that were tested in the 3 iterations of the design stage covers 4 main categories: (1) functionality of the QoL-ME, (2) navigation, (3) personalization, and (4) appearance.

**Figure 2:**
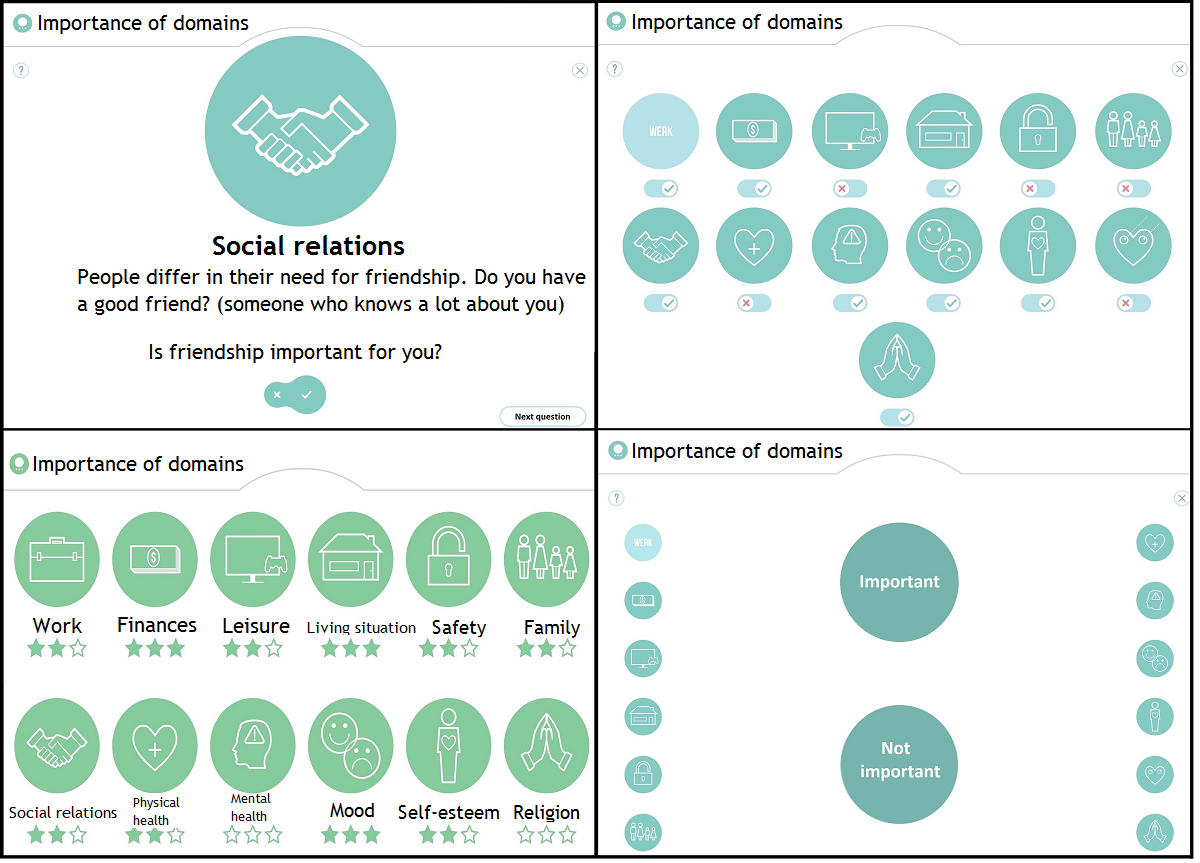
Four mechanisms for selecting additional modules. In the top-left mechanism, users rate the importance of every domain individually. In the top right corner, the same mechanism is displayed for every domain at the same time. In the bottom left panel, every domain is rated by giving it one to three stars. In the bottom right panel, the icons on the left and right have to be swiped or dragged to one of the two circles.

First, participants commented on the functionality of the QoL-ME. Specifically, these comments were related to different forms of interaction, operating the app, and receiving feedback. Several mechanisms for selecting the additional modules of the QoL-ME were tested. Figure 2 displays 4 of these possible modes of interaction. Please note that as the QoL-ME is developed for use in the Netherlands, it contains some Dutch text. To improve the clarity of the screenshots that are part of Figure 2 and other figures, any Dutch text has been translated to English.

**Table 1:** Basic demographic characteristics of participants per iteration of the development of the QoL-ME.

Stage of development and iteration number		Participants (n)		Male participants, n (%)		Age, mean (SD)	
**Brainstorming stage**							
	Iteration 1		10		7 (70)		34.2 (12.8)
**Design stage**							
	Iteration 2		8		7 (88)		32.8 (13.6)
	Iteration 3		8		7 (88)		38.9 (12.8)
	Iteration 4		9		8 (89)		41 (14.9)
	Total		25		22 (88)		37.7 (14.3)
**Usability stage**							
	Iteration 5		9		6 (67)		42 (17.5)
	Iteration 6		15		13(87)		49.7 (11.3)
	Total		24		19 (79)		46.8 (14.4)
Total entire development		59		48 (81)		40.8 (15)	

**Table 2:** Overview of the feedback obtained during the 3 iterations of the design stage of the development of the QoL-ME.

Category and subcategory		Feedback
**Functionality**		
	Interaction: selecting additional modules	Swiping icons preferred in domain selection
	Interaction: items additional modules	Visual analog scale preferred to answer questions of additional modules
	Input	Most participants had no difficulty with the touchscreen, but some did: enable alternative options such as a keyboard and mouse
	Feedback	Simple visualization of results, avoiding graphs
**Navigation**		
	Linear structure	Inevitable choices in hierarchical structure were confusing: preference for linear structure
	Confirming choices	Confirmation of choices (next and previous) was appreciated
	Size and position of buttons	Large buttons with fixed sizes (bottom left and bottom right of screen)
**Personalization**		
	Creating user profiles	Too much effort and no added value
	Personalization of background and colors	No added value
**Appearance**		
	Layout	Calm and professional layout was evaluated positively
	Font size	Large font was advised
	Contrasts	Sufficient contrast between text and background

**Figure 3:**
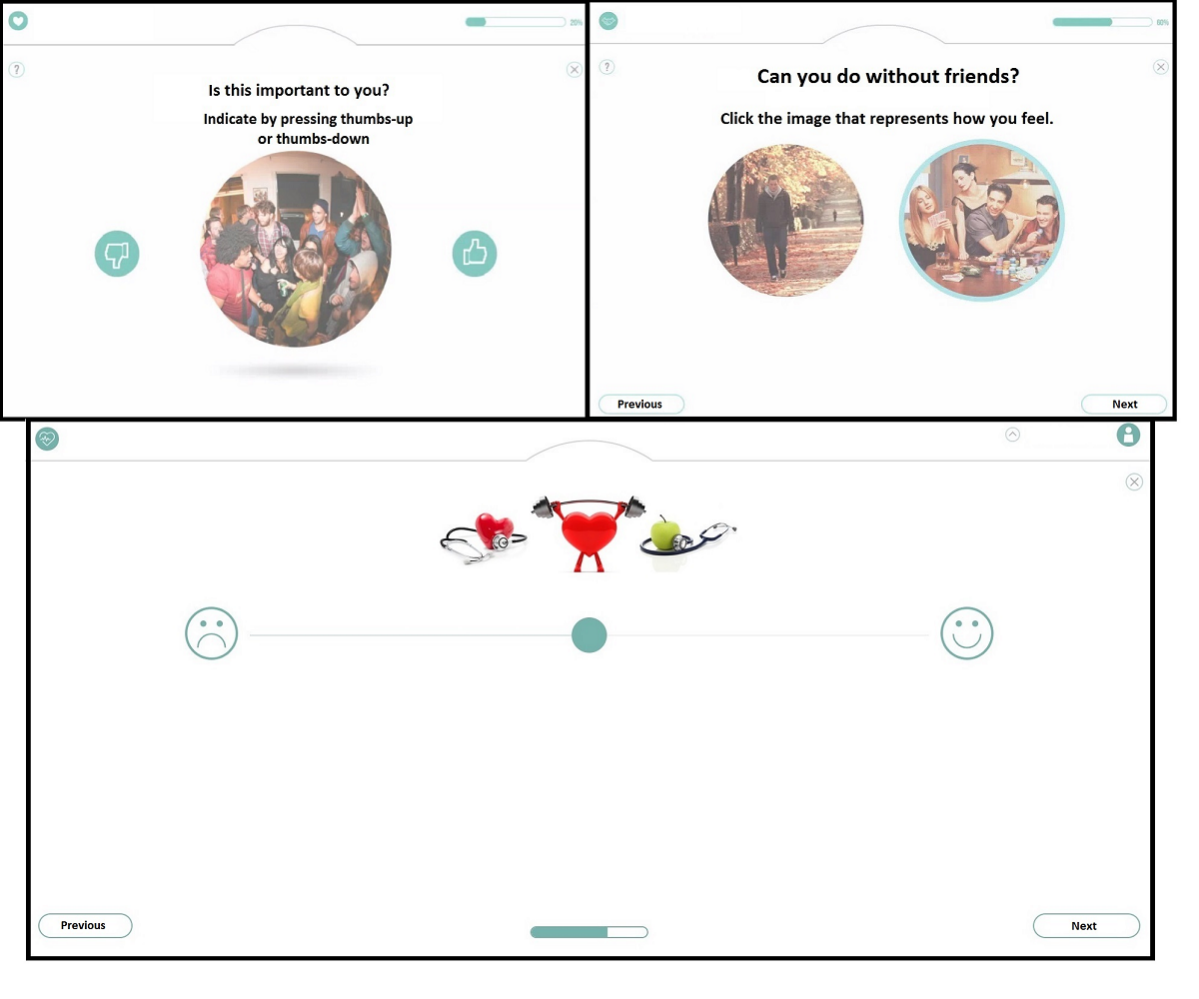
Three possible mechanisms for interaction in the items of the additional modules.

Participants indicated a strong preference for the option in which icons had to be swiped (see the bottom right panel in Figure 2). In addition, multiple forms of interaction for use in the items of the additional modules of the QoL-ME were tested. Figure 3 provides an overview of 3 interaction mechanisms. As participants indicated a preference for VAS, VAS was used in the prototype. The majority of participants had little to no difficulty with the touchscreen, even though some participants initially described themselves as computer illiterate and reported never having used a touchscreen before. Some participants did indicate that it would be a good idea to also enable the use of a keyboard and mouse to operate the QoL-ME.

**Figure 4:**
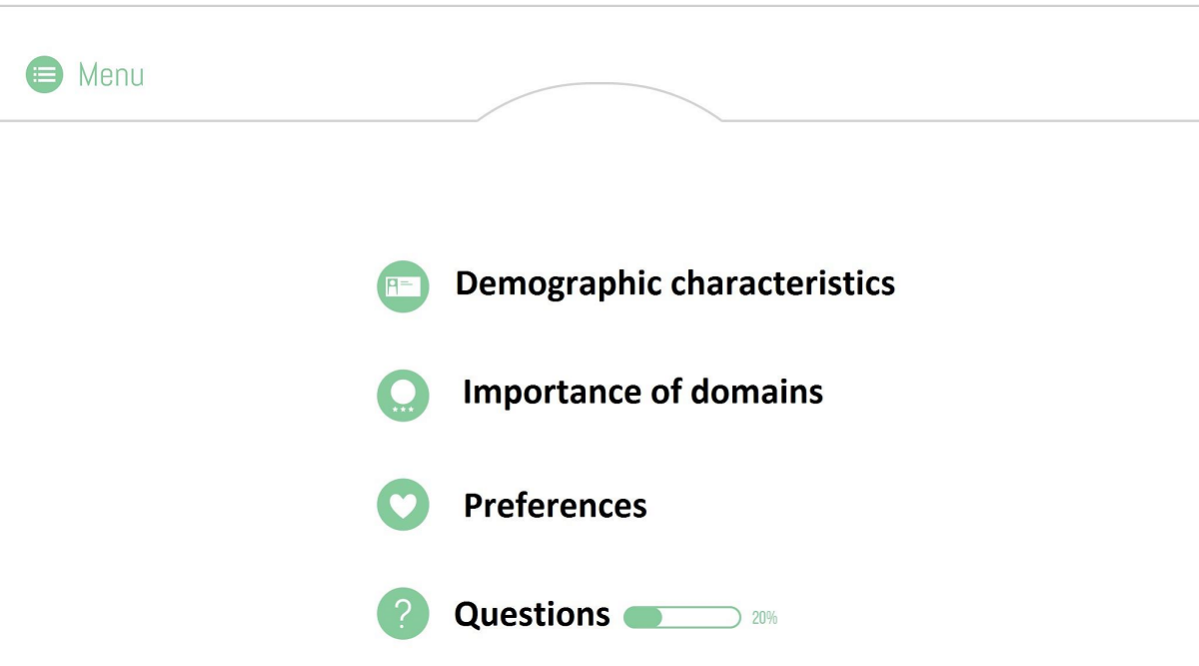
Earlier versions of the QoL-ME required users to select 1 of 4 menu options.

Second, initial versions of the QoL-ME allowed participants to select the order in which they wanted to progress through the app. Participants had the opportunity to choose 1 of the 4 menu items (see Figure 4).

**Figure 5:**
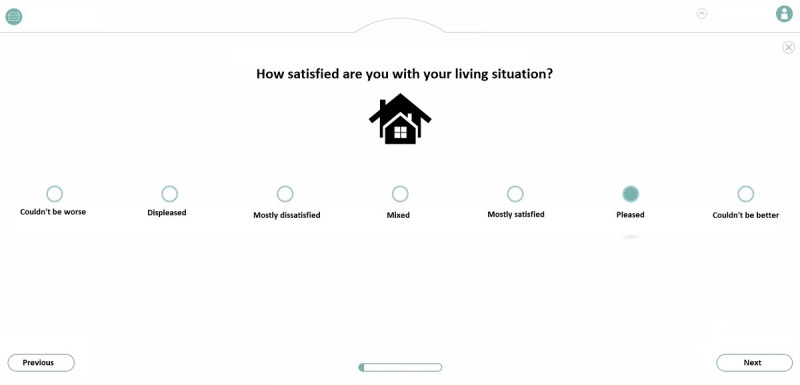
Users are required to navigate explicitly by selecting 1 of the 2 buttons at the bottom left and bottom right of the screen.

Most of the participants in the design stage were unsure which of the 4 options to select and preferred a linear navigational structure, which was adopted in later versions of the QoL-ME. The QoL-ME requires participants to navigate the app explicitly by selecting buttons at the bottom of the screen (see Figure 5). Participants saw this as a valuable feature, as it allowed them to correct possible mistakes before progressing to the next item or part of the app and because it introduces predictability. In addition, participants indicated a preference for large navigation buttons with a fixed location.

**Figure 6:**
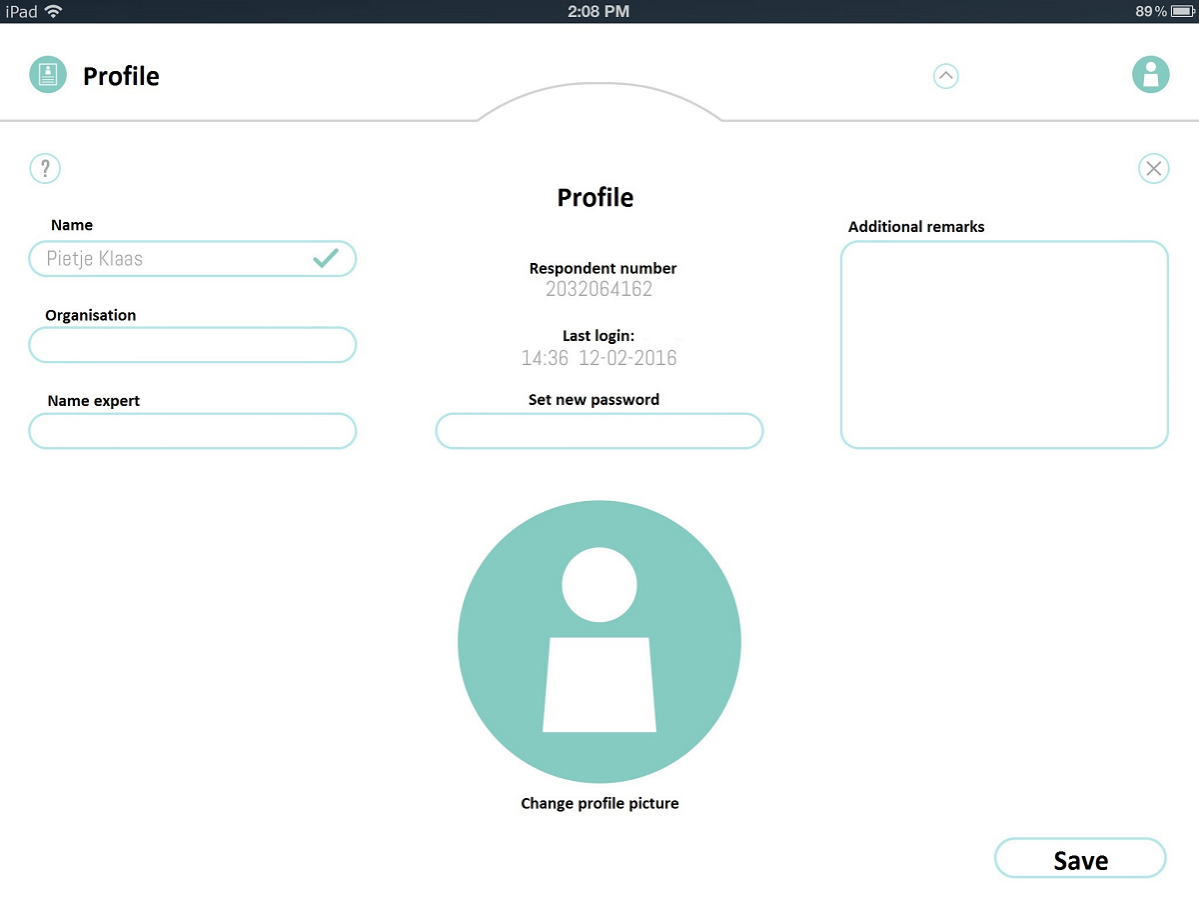
Early versions of the QoL-ME included the possibility to create a user profile.

**Figure 7:**
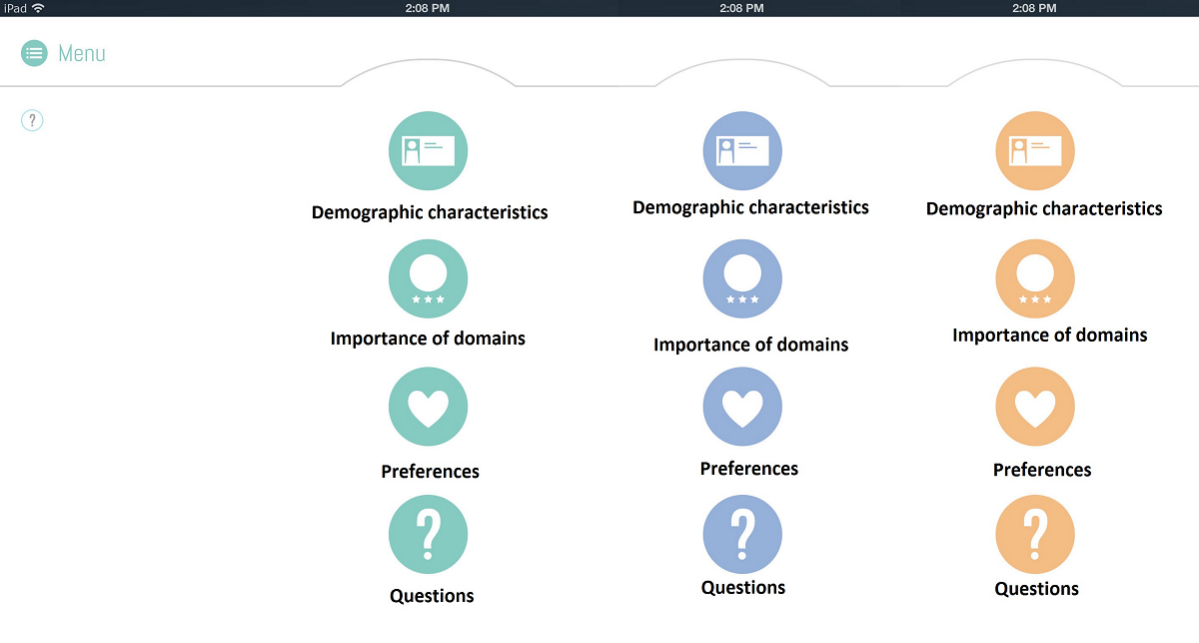
Earlier versions of the QoL-ME allowed users to customize the colors of the QoL-ME.

Third, possibilities for the personalization of the QoL-ME were explored. Versions of the QoL-ME that were tested in this stage allowed participants to create a user profile (see Figure 6) and to select 1 of the several colors for the layout of the app (see Figure 7). However, participants were not enthusiastic about these features, and they were dropped in later versions of the QoL-ME.

Fourth, throughout the design stage, participants had a fondness for the calm and clean layout of the QoL-ME (see Multimedia Appendices 2 and 3). Several participants noted that the layout of the QoL-ME made it look professional and added to its credibility and trustworthiness. However, 2 participants found the QoL-ME’s appearance to be a little dull. In addition, participants preferred large fonts and sufficient contrast between text and background.

The average SUS score was 76.8 (SD 14.9; median=76.3), and scores ranged between 35 and 97.5. According to the classification reported by Bangor and Kortum [50], a SUS score of 76.8 indicates good to excellent usability.

After filling out the SUS, participants were invited to share any additional feedback. Most of the participants in the usability stage did not have any additional feedback and found the QoL-ME to be easy to use, as reflected by their SUS scores. Some participants wanted more explanation on how to select the content of the additional modules of the QoL-ME. Others had difficulty in placing the VAS exactly at the halfway point. These minor remarks were taken into consideration, and some slight modifications to the prototype were made, resulting in the QoL-ME that is described in the following section.

### Final QoL-ME

The following section contains a brief walkthrough of the QoL-ME. An accompanying video is provided in Multimedia Appendix 2. After logging into the QoL-ME app using their email address and a personalized password, users arrive at the home screen, which includes a brief explanation of the goal and structure of the app. Users have the opportunity to view a short video tutorial in which the structure and operating mechanisms of the QoL-ME are explained (see Multimedia Appendix 2). After pressing the *start* button on the homepage, users arrive at the core version of the QoL-ME. To determine which of the 2 core versions is applicable to the user, users are first requested to indicate whether they consider themselves as being homeless or not.

**Figure 8:**
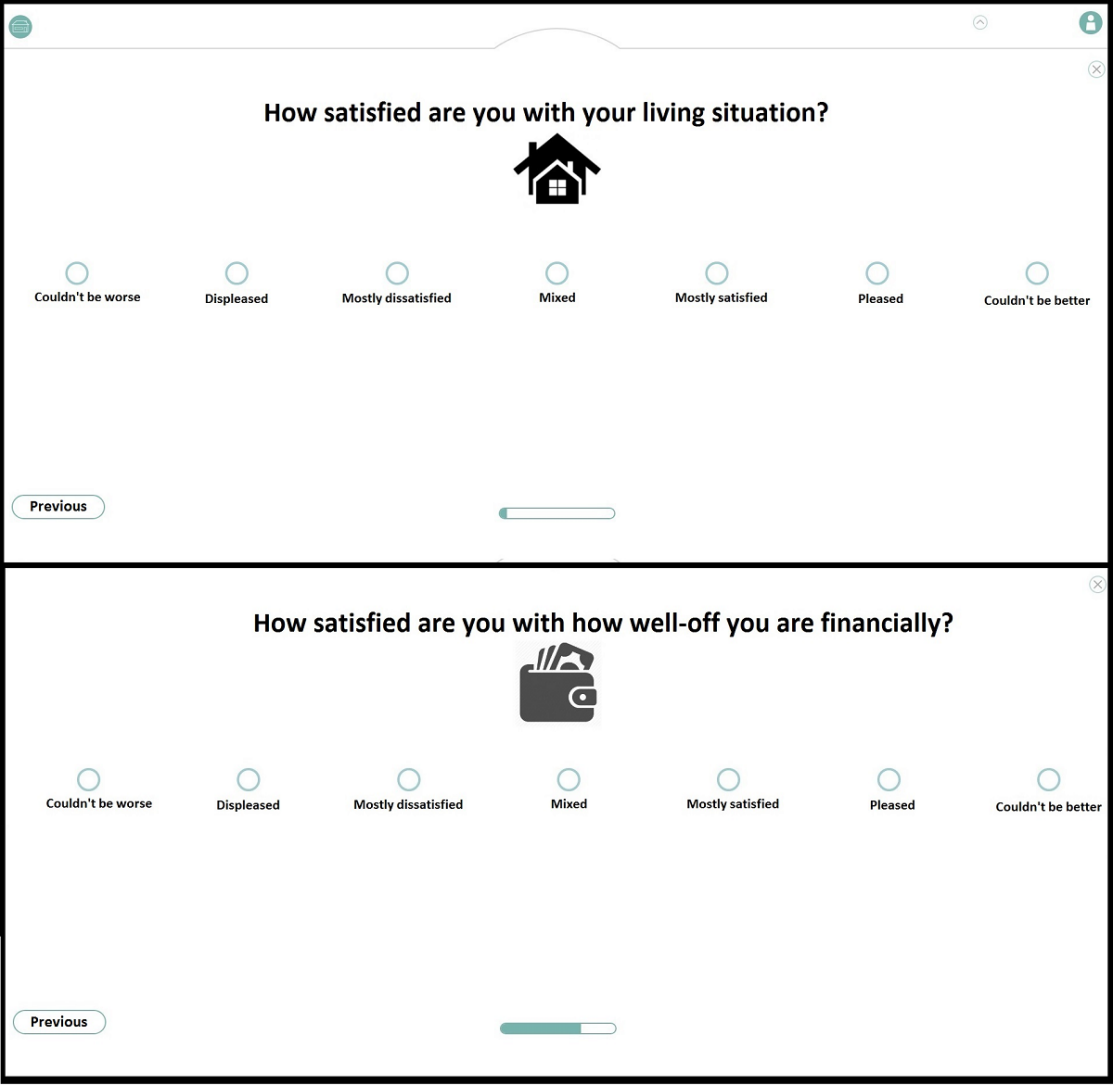
Examples of 2 items of the QoL-ME’s core version.

An affirmative answer will lead the user to the core version for people who are homeless. Alternatively, users are invited to fill out the core version for people with psychiatric problems. Having selected the appropriate core version, users arrive at an explanation of the core version. Examples of 2 items of the core version are presented in Figure 8.

**Figure 9:**
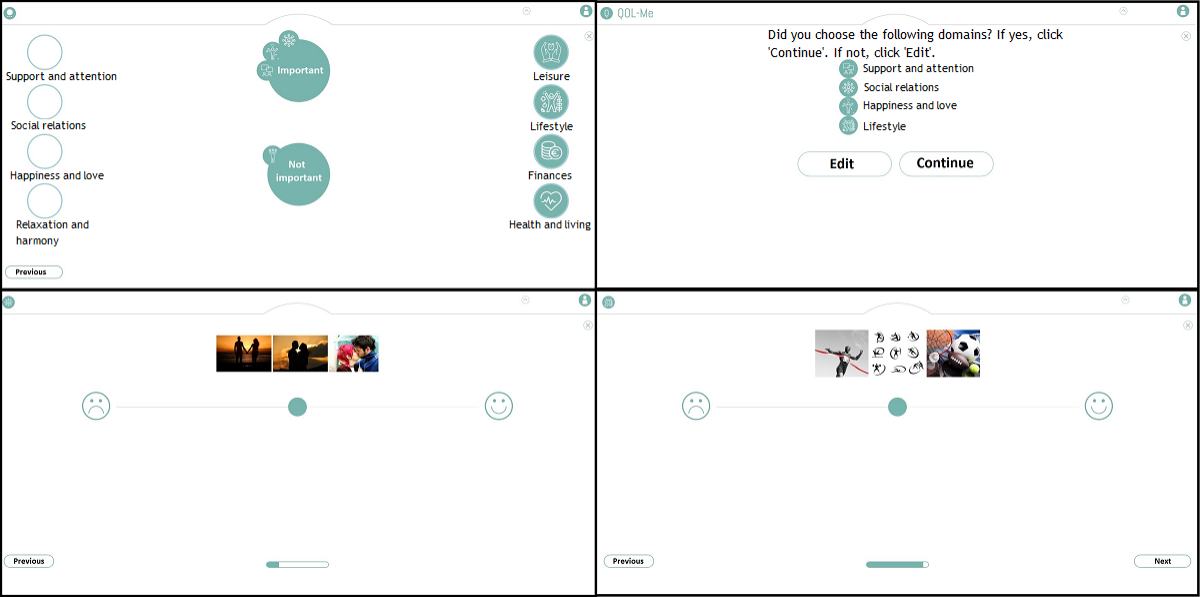
Four screenshots depicting the additional modules of the QoL-ME. The top left panel displays the mechanism for selecting additional modules. Respondents are invited to drag 8 icons, corresponding to the 8 modules, to either a circle that says important or a circle that says not important. The top right panel shows how respondents are asked to confirm their choice of additional modules. The 2 remaining panels provide examples of items of the additional modules.

Having completed the core version, users are asked to indicate which domains of the additional modules are important to them. A screenshot of the mechanism used to select add-on modules is available in Figure 9. To ensure that the correct domains have been selected, users are asked to confirm their choice (see Figure 9).

**Figure 10:**
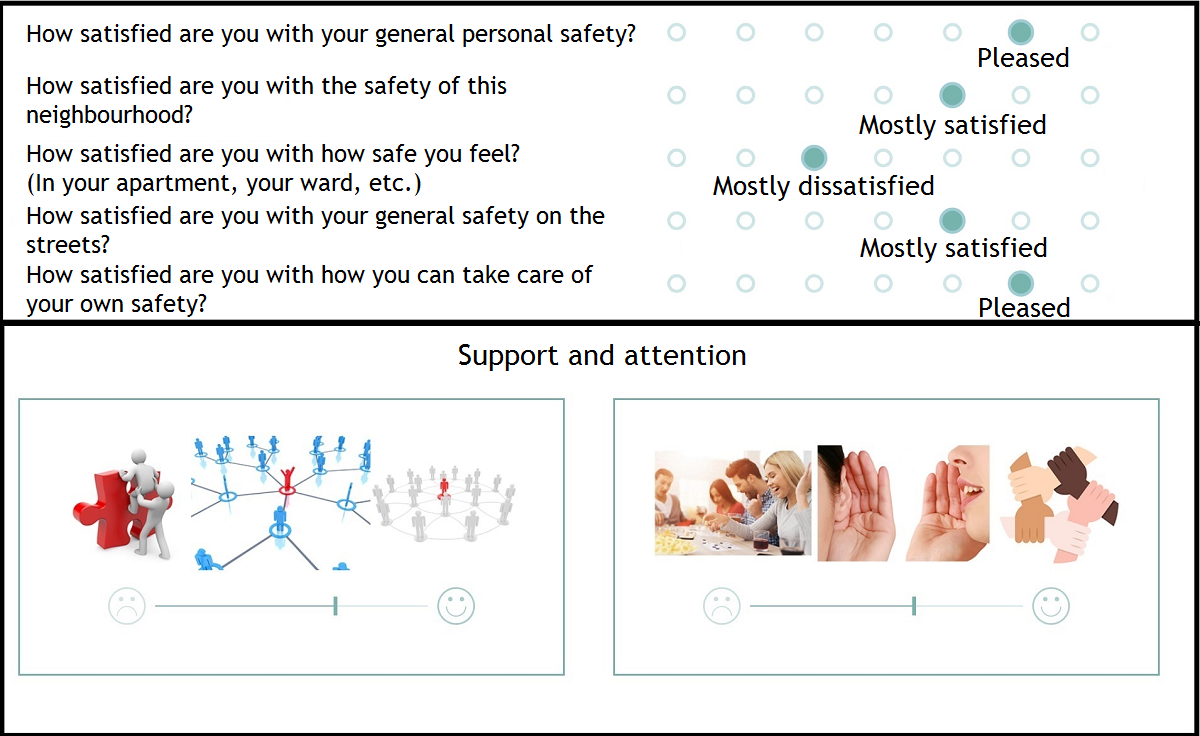
Results section of the QoL-ME. The top panel displays how the results of the core version are displayed, whereas the bottom panel demonstrates the results of the additional modules.

Next, users answer questions corresponding to their selection of additional modules. Figure 9 provides examples of 2 visual items of the additional modules. Once all questions have been answered, users have the option to review their answers on the results page (see Figure 10).

## Discussion

### Principal Findings

This study pertains to the cocreative development of the QoL-ME: an innovative, personalized, and visual QoL assessment app. A diverse group of people with severe mental health problems contributed to every iteration of the development. The feedback regarding the design and functionality of the QoL-ME that was provided by participants played an essential and central role in the development. The usability evaluation using the SUS revealed good to excellent usability of the QoL-ME.

The feedback gathered during the development of the QoL-ME can be split into 3 categories: (1) feedback that corresponds to previous design recommendations [21,28,31], (2) feedback that deviates from these recommendations, and (3) findings specific to the QoL-ME and its function as a visual QoL assessment app. First, some of the feedback received in the design stage corresponds to existing recommendations reported by Rotondi et al [28] as part of their FEDM and by Bernard et al [21] in their review of factors that facilitate the Web usage of people with mental disorders. The majority of participants had little difficulty in operating the touchscreen. However, some participants recommended enabling the use of a keyboard and mouse. These findings correspond to the results by Bernard et al [21], who recommend providing multiple, alternative ways to operate a technology. Moreover, the fixed position of the navigation buttons made using the QoL-ME predictable and clear, which was in line with recommendations included in the FEDM [28]. In addition, participants were positive regarding the appearance of the QoL-ME and experienced it as calming, pleasant, and professional. Furthermore, participants stressed the importance of using sufficient contrasts between important elements and the background of the apps and of using large fonts. These findings regarding the layout, font size, and contrasts of the QoL-ME confirm existing recommendations [21,28].

Second, some feedback deviated from the design guidelines found in the literature. Of the main recommendations of the FEDM 1 covers the navigational structure of a digital technology. The FEDM advocates a shallow hierarchical structure, whereas participants in this study strongly preferred a linear structure, as it removed the need for making navigational choices. Furthermore, the FEDM promotes scrolling down a page for additional content over paging: having to go to another page for additional content. However, in this study, participants indicated a clear preference for paging over scrolling. The fact that the FEDM primarily targets websites, whereas the QoL-ME is an app, may explain this deviation. General guidelines that target smartphone apps specifically do recommend minimizing navigational choices and advise against scrolling [57]. An alternative explanation for the deviating findings lies in the increasing importance and usage of digital technologies, which may cause shifts in user preferences. In addition, Bernard et al [21] identified the personalization of the appearance of a digital technology, including color and font size, as a facilitating factor. In this study, participants did not welcome the possibilities for personalization included in earlier versions of the QoL-ME. Possibly, the personalization of the appearance of the QoL-ME was seen as a distraction as it was unrelated to the function of the QoL-ME.

Third, 2 preferences indicated by participants are specific to the functionalities of the QoL-ME and are, therefore, unrelated to existing design recommendations. First, participants preferred the use of VAS scales over the Likert scale to answer the items of the additional modules. This finding confirms earlier research [58]. Second, participants preferred a mechanism that involved swiping or dragging icons for the selection of the additional modules. Both mechanisms were tested on a touchscreen device, which may have enhanced their popularity. Prior research confirms the accessibility of a touchscreen-based interaction [59,60].

Usability evaluations of the QoL-ME using the SUS reveal good to excellent usability. The average SUS score of 76.8 obtained in this study is similar to SUS scores gathered in usability evaluations of comparable apps. Kooistra et al [61] evaluated the usability of a blended cognitive behavioral therapy for people with depression and found an average SUS score of 73.2. Furthermore, Fiorillo et al [62] obtained an average SUS score of 81.8 when evaluating the usability of a Web-based acceptance and commitment therapy intervention for people with trauma-related psychological difficulties. In addition, Kobak et al [63] reported an average SUS score of 83.5 in their evaluation of computerized cognitive behavior therapy for people with obsessive-compulsive disorder.

### Strengths and Limitations

The diversity of the study population is an important strength. Participants from diverse age groups and care backgrounds shared their insights regarding the QoL-ME. This diverse sample ensures that the QoL-ME appeals to a large and diverse group of potential users and may enhance the generalizability of the results to people with severe mental health problems. The strong emphasis on collaboration with people with severe mental health problems can be seen as another strength [36]. People with severe mental health problems were heavily involved in the development of the QoL-ME, and their feedback, tips, and insights strongly influenced the direction of the development.

Apart from these strengths, several limitations ought to be taken into account when interpreting the results of this study. First, the sample was not selected randomly but by a combination of convenience sampling and stratified sampling. This sampling strategy may negatively affect the generalizability of the results. At the same time, the aforementioned diversity of the sample indicates that the negative consequences of the sampling strategy are minimal. Second, the results may be biased by a selection effect. It is likely that clients who were interested in this study had at least some affinity and experience with digital technology and apps. If this is the case, potential issues in the design of the QoL-ME may not have been uncovered. However, a number of participants described themselves as digital illiterates and some even indicated never having used apps or touchscreen devices before. This anecdotal evidence appears to indicate that no major selection effect occurred. Nevertheless, participants’ previous experience with digital technologies was not investigated systematically, and therefore, no firm conclusion can be drawn. Third, the group of participants who evaluated the usability of the QoL-ME using the SUS was rather small. However, a study by Tullis and Stentson [64] revealed a sample of 12 to 14 participants is sufficient to obtain reliable results using the SUS. A fourth limitation concerns the dearth of available information regarding the background of participants. However, in this study, we strove to include a broad group of participants so that the QoL-ME suits a sample of people with severe mental health problems with diverse vulnerabilities and problems. Therefore, no conclusions regarding the appropriateness of the QoL-ME for groups with specific cultural backgrounds, psychopathology, or socioeconomic status can be drawn on the basis of this study. Further research will have to reveal whether the cocreative development has resulted in an app that is suitable for specific groups.

### Conclusions

The cocreative development of the QoL-ME resulted in an innovative, personalized, and visual app for QoL assessment. Overall, participants had little difficulty in operating the QoL-ME and were positive regarding its usability. Participants indicated a preference for paging over scrolling, linear navigation, a clean and minimalist layout, and the use of touchscreen functionality in various modes of interaction. Further research is needed to evaluate both the validity and reliability of the QoL-ME. In addition, it is important to investigate the usefulness of the QoL-ME for both clients and care professionals in practice. Moreover, for the QoL-ME to be of use in clinical practice, its sensitivity to change in QoL ought to be examined.

## Appendix

Multimedia Appendix 1 Topic list used during the brainstorming stage of the development of the QoL-ME.

Multimedia Appendix 2 Short video introducing the QoL-ME. Users may choose to watch the video directly after logging into the QoL-ME.

Multimedia Appendix 3 Video walkthrough of the QoL-ME.

## Abbreviations

eHealth: electronic health
FEDM: Flat Explicit Design Model
LQoLP: Lancashire Quality of Life Profile
QoL: quality of life
SUS: System Usability Scale
VAS: visual analog scale
