# Supplementary material for: Cocreative Development of the QoL-ME: A Visual and Personalized Quality of Life Assessment App for People With Severe Mental Health Problems
Source: JMIR Ment Health. 2019 Mar 28;6(3):e12378. doi: 10.2196/12378 (PMC6458539; doi:10.2196/12378)
Supplement: Multimedia Appendix 1 [file mental_v6i3e12378_app1.pdf]

Multimedia Appendix 1. Topic list brainstorm stage

Topic list brainstorm stage

The questions used to elicit information in the brainstorm stage can be split into four categories: 1) participants' experience with smart devices and apps, 2) participants' experience with (quality of life)-questionnaires, 3) participants' ideas regarding the QoL-ME, and 4) participants' reactions to the basic initial designs.

1. **Questions regarding experiences with smart devices and apps.**

- Do you own or use a smartphone or tablet?
- If yes, what do you use it for?
- Do you have any apps installed? If yes, which apps? If not, why not?
- For what purpose do you use these apps?
- What do you like about these apps? Are there any characteristics of these apps that you particularly like? And any characteristics that you do not like at all?
- Do you often make use of apps or websites that require you to login using a username/password combination?
- Do you trust the people behind the app?
- What makes that you trust or distrust an app?

2. **Questions regarding experiences with (quality of life) questionnaires**

- Do you have experience filling out quality of life questionnaires? And if not, do you have experience with other questionnaires?
- What do you think of these questionnaires? What do you like or dislike?
- Can you think of ways in which these questionnaires can be improved?
- How would you like to assess your quality of life?

3. **Ideas regarding the QoL-ME**

- We are developing a digital quality of life assessment app. Can you think of reasons for why you would not want to use this app?
- And are there reasons for why you would want to use the app?

4. **Questions regarding the basic initial designs**

- **[Login screen]** Is it clear what you need to do to access the application? If not, why not?
- **[Menu screen]** What do you think you need to do here?
- **[Testing possible modes of interaction]** What would you do if confronted with this screen?
- **[Mock pictures used in the initial designs]** What do you think these pictures depict?
- Do you think that this app is something you would want to use when finished? If yes, why? If not, why not?
